# Comparison of the Supreme™ and ProSeal™ laryngeal mask airways in infants: a prospective randomised clinical study

**DOI:** 10.1186/s12871-017-0418-z

**Published:** 2017-09-05

**Authors:** Sibel Oba, Hacer Sebnem Turk, Canan Tulay Isil, Huseyin Erdogan, Pinar Sayin, Ali Ihsan Dokucu

**Affiliations:** 1 Department of Anaesthesiology and Reanimation, Sisli Hamidiye Etfal Education and Research Hospital, Halaskargazi Cad., Etfal Sok, 34377 Istanbul, Turkey; 2 Department of Paediatric Surgery, Sisli Hamidiye Etfal Education and Research Hospital, Halaskargazi Cad., Etfal Sok, 34377 Istanbul, Turkey

**Keywords:** Laryngeal mask airway ProSeal, Laryngeal mask airway Supreme, Infants

## Abstract

**Background:**

The Supreme™ and ProSeal™ laryngeal mask airways (LMAs) are widely used in paediatric anaesthesia; however, LMA use in infants is limited because many anaesthesiologists prefer to use tracheal intubation in infants.

In this study, we compared the Supreme and ProSeal LMAs in infants by measuring their performance characteristics, including insertion features, ventilation parameters, induced changes in haemodynamics and rates of postoperative complications.

**Methods:**

Infants of ASA physical status I scheduled for elective, minor, lower abdominal surgery were divided into two groups: the Supreme LMA group and the ProSeal LMA group. Times and ease of LMA insertion were noted. The percentages of tidal volume leakage as well as peak, mean and leakage pressures for all infants were measured. Heart rate (HR), oxygen saturation (SpO2) and end tidal carbon dioxide (EtCO2) values were recorded before and after LMA insertion and before and after extubation. After extubation, complications and adverse effects were noted.

**Results:**

Demographic and surgical data were similar between the two groups. LMA insertion times were shorter for the ProSeal group than for the Supreme group (*P* < 0.002). The mean HR value for the ProSeal group was lower than for the Supreme group (*P* < 0.011). Both the peak pressure and the leakage percentage for the ProSeal group were statistically lower than for the Supreme group. The leakage pressure for the ProSeal group was statistically higher than for the Supreme group (*P* < 0.001).

**Conclusions:**

The ProSeal LMA is superior to the Supreme LMA for use in infants due to the ease of insertion, high oropharyngeal leakage pressure and fewer induced changes in haemodynamics.

**Trial registration:**

ClinicalTrial.gov, NCT03251105, retrospectively registered on 15 Aug 2017.

## Background

The use of laryngeal mask airways (LMAs) for modern paediatric anaesthesia has gained popularity. In children, the use of LMAs decreases complications after anaesthesia because neuromuscular blockade is not required [1]; however, in infants younger than 1 year of age, LMA insertion is not easily accomplished due to the developing airway anatomy and fragility of the oesophageal mucosa. Studies evaluating LMA use in such a young population are limited because many anaesthesiologists still prefer to use tracheal intubation in infants [2–8].

In the current paediatric literature, different LMA models have been evaluated; however, these studies were performed on a wide range of children, from infants to 18-year-olds [9, 10]. Consequently, it has been difficult to evaluate the results of these studies for LMA use in infants alone.

Among the different LMAs available, the Supreme™ and ProSeal™ LMAs are the most frequently used for paediatric anaesthesia. A gastric access canal has been incorporated into the second-generation devices; this gastric access canal allows for gastric venting.

In this study, we compared the Supreme and ProSeal LMAs in an infant population by measuring their performance characteristics, including insertion time, insertion success, airway leak pressure, induced changes in haemodynamics, oxygen saturation (SpO_2_) and end tidal CO_2_ (EtCO_2_) values, and postoperative complications.

## Methods

This prospective randomised study was approved by the Governmental Ethics Committee of Sisli Hamidiye Etfal Education and Research Hospital (SEEAH/26.04.2016/659) and retrospectively registrated at the Clinical Trials Protocol Registration and Result System with the Identification Number NCT03251105/15.08.2017; verbal and written informed consent was obtained from the parents of all infants participating in the study.

Children of American Society of Anaesthesiologists (ASA) physical status I who were younger than 12 months of age and were scheduled for elective, minor (< 1 h duration), lower abdominal surgery, including unilateral herniorrhaphy and unilateral orchidopexy, were enrolled in this randomised study. Exclusion criteria were premature birth, potentially difficult airway, clinically significant upper respiratory tract infection and risk of aspiration, such as gastro-oesophageal reflux disease.

**Fig. 1:**
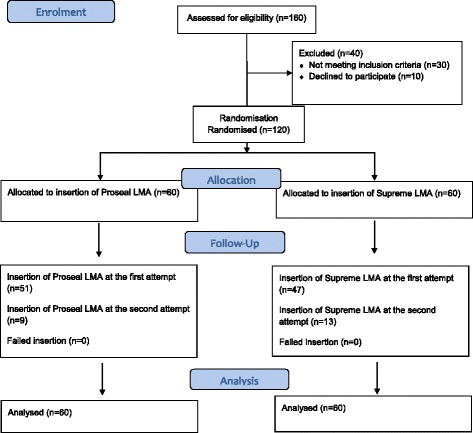
CONSORT flow diagram

A total of 120 infants (Fig. 1) were randomly assigned to either the ProSeal group (Group P; *n* = 60) or the Supreme group (Group S; *n* = 60). A random-number sequence was created for group assignments by a nurse who did not participate in either the anaesthesia care or the outcome assessments.

No medications were administered before inducing anaesthesia. Infants in the operating room were monitored using pulse oximetry, electrocardiography, noninvasive blood pressure measurements, capnography and monitoring of the bispectral (BIS) index. Patient demographics, such as sex, age (in months), surgery type and duration, and duration of anaesthesia, were recorded. The same type of anaesthesia was used in both groups.

The induction of anaesthesia was induced by inhalation of 6% sevoflurane via a face mask. After insertion of an intravenous cannula, 1 μg/kg fentanyl and 1% propofol were administered until the BIS index was less than 60. The size of each LMA was selected in accordance with the manufacturer’s guidelines using the patient’s weight (size 1 for infants weighing less than 5 kg and size 1.5 for infants weighing between 5 and 10 kg). LMA cuffs were insulated to obtain a pressure between 30 and 40 mmHg, measured using a Mallinckrodt. LMAs were inserted by the same experienced anaesthetist who had previously inserted these devices on at least 50 occasions. A successful LMA insertion was confirmed by observing bilateral chest wall movement and a square capnography waveform. Two attempts were made to insert the LMAs in both groups. Insertion time was measured from the moment an LMA was selected until the appearance of the first waveform on the capnogram. The ease of insertion was assessed using a grading score of 1–3 (1: easy; 2: difficult; 3: impossible) [11, 12]. A failed insertion was defined as no successful insertion of the LMA after two attempts.

For the required effect of anaesthesia, 6% sevoflurane in a 50% oxygen–air mixture was used to maintain a BIS index between 50 and 60. All patients were mechanically ventilated using a Drager anaesthesia machine (Primus), with maintenance of a tidal volume between 6 and 8 mL/kg, a respiratory frequency between 18 and 24, and an EtCO_2_ value less than 50.

No neuromuscular blocking agents were administered.

The percentage of tidal volume leakage and the peak, mean and leakage pressures for all patients were measured thrice. The first measurement was performed immediately after the LMA insertion, the second measurement was performed 10 min after LMA insertion and the third measurement was performed before extubation. Mean values were recorded for all measurements. To calculate the leakage percentage, the expiratory tidal volume was deducted from the inspiratory tidal volume, and the result was divided by the inspiratory tidal volume. The leakage pressure was determined by closing the expiratory valve of the circle system at a fixed gas flow of 3 L/min and recording the airway pressure when equilibrium was reached. While performing manual positive pressure ventilation, the maximum peak pressure that caused air leakage from the mouth (audible) was accepted as the leakage pressure [2, 12].

The HR, SpO_2_ and EtCO_2_ values were recorded for each patient before and after LMA insertion and before and after extubation.

At the end of surgery, the LMA was removed when the patient regained consciousness. After extubation, any mucosal hyperaemia, mucosal damage or blood on the surface of the LMA was recorded. Any adverse effects, such as cough, bronchospasm, desaturation, gastric dilatation or vomiting, were noted.

We calculated the sample size as 40 patients in each group, with the assumption of at least 30% possible difference between any two groups. Therefore, we allocated 60 patients into each group to obtain an alpha error of 5% and statistical power of 80% while considering possible insufficient data collection.

SPSS 15.0 for Windows was used for the statistical analyses. Descriptive statistics are provided as the number and percent changes for categorical variables and as the mean, standard deviation and median for numerical variables. For numerical variables, the Student’s *t*-test was used in cases of normal distribution, and the Mann–Whitney U test was used in other cases. The ratio of categorical variables between groups was analysed using a chi-square test. A *P*-value of less than 0.05 was considered statistically significant.

## Results

In terms of demographics and surgical data, no differences were found between the ProSeal and Supreme groups (Tables 1 and 2).

**Table 1:** Patient demographics

		Group P (*n* = 60)	Group S (*n* = 60)
Age (months) mean ± SD		5.7 ± 3,2	5.9 ± 3,0
Gender (F/M) n (%)		14 (23.3)/46 (76.7)	18 (30.0)/42 (70.0)
Weight (kg) mean ± SD		7,3 ± 2,0 (7)	7,7 ± 2,2 (7)
LMA Size 1	n (%)	20 (33.3)	18 (30)
1.5	n (%)	40 (66.7)	42 (70)

*F* female, *M* male, *SD* standard deviation

**Table 2:** Surgical characteristics

	Group P (*n* = 60)	Group S (*n* = 60)	*P*
	Mean ± SD	Mean ± SD	
Anaesthesia Time (min)	49.1 ± 18,3	48.6 ± 14,1	0.696
Surgical Time (min)	37.8 ± 15,5	39.1 ± 14,2	0.359
LMA Duration (min)	44.8 ± 18,0	44.0 ± 14,3	0.729
Recovery Time (min)	7.3 ± 2,0	7.6 ± 2,2	0.274

*Min* minutes, *LMA* laryngeal mask airway; *P* < 0.05 is statistically significant

The insertion time of the ProSeal group was shorter (8.1 ± 2.9 min) than in the Supreme group (8.8 ± 2.9). This difference was statistically significant (*P* < 0.002).

In the ProSeal group, the LMA was successfully inserted in 51 patients (85%) after a single attempt versus 47 patients (78.3%) in the Supreme group; in 9 patients (15%) in the ProSeal group and 13 patients (21.7%) in the Supreme group, insertion of the LMA was successful in the second attempt. There were no failed insertions. The ease of insertion was similar in both groups (Table 3).

**Table 3:** Laryngeal Mask Airway (LMA) insertion characteristics

		Group P (*n* = 60)	Group S (*n* = 60)	*P*
LMA Insertion Time (sec) mean ± SD		8.1 ± 2,9	8.8 ± 2,0	0.002
Number of Manipulations Required to Insert LMA n (%)	1	51 (85.0)	47 (78.3)	0.345
	2	9 (15.0)	13 (21.7)	
Ease of LMA Insertion n (%)^a^	1	51 (85.0)	46 (76.7)	0.246
	2	9 (15.0)	14 (23.3)	
	3	0	0	

*LMA* laryngeal mask airway, *SD* standard deviation, *sec* seconds; *P* < 0.05 is statistically significant; ^a^ease of LMA insertion was graded as 1: no resistance (easy), 2: moderate resistance (difficult) or 3: inability (impossible) to place the LMA

The pre- and post-extubation HR values were statistically lower in the ProSeal group than in the Supreme group (*P* < 0.011 and *P* = 0.042, respectively).

The SpO_2_ and EtCO_2_ values were not statistically different between the two groups (Table 4).

**Table 4:** Haemodynamic variables

	Group P (*n* = 60)	Group S (*n* = 60)	*P*
	Mean ± SD	Mean ± SD	
HR Beginning	143.4 ± 9.8	144.9 ± 6.4	0.309
HR after LMA Insertion	140.3 ± 11.5	140.9 ± 8.4	0.779
HR before Extubation	130.6 ± 9.8	135.4 ± 6.6	0.011
HR after Extubation	135.4 ± 8.3	138.9 ± 5.7	0.042
SpO_2_ Beginning	99.9 ± 0.3	100.0 ± 0.2	0.698
SpO_2_ after LMA Insertion	99.3 ± 1.4	99.1 ± 1.2	0.262
SpO_2_ before Extubation	99.6 ± 0.7	99.4 ± 0.9	0.126
SpO_2_ after Extubation	99.6 ± 0.8	99.6 ± 0.6	0.243
EtCO_2_ Beginning	32.1 ± 1.8	32.3 ± 1.8	0.603
EtCO_2_ before Extubation	32.3 ± 1.8	32.3 ± 1.8	0.879

*HR* heart rate, *LMA* laryngeal mask airway, *SpO*_*2*_ oxygen saturation, *EtCO*_*2*_ end tidal carbon dioxide value; *P* < 0.05 is statistically significant

The peak and mean pressures, as well as the leakage percentage, of the ProSeal group were lower than in the Supreme group; these differences were statistically significant.

The leakage pressure of the ProSeal group was higher than in the Supreme group; this difference was statistically significant (Table 5).

**Table 5:** Mean ventilation parameters^a^

	Group P (*n* = 60)	Group S (*n* = 60)	*P*
	Mean ± SD	Mean ± SD	
Peak Pressure (cm H_2_O)	18.1 ± 3.2	20.1 ± 2.9	**< 0,001**
Mean Pressure (cm H_2_O)	7.3 ± 1.2	7.8 ± 1.1	**0,011**
Leakage Percentage (%)	6.4 ± 1.3	7.3 ± 1.0	**< 0,001**
Leakage Pressure (cm H_2_O)	33.0 ± 2.2	31.5 ± 1.8	**< 0,001**

*SD* standard deviation; *P* < 0.05 is statistically significant; ^a^these parameters were measured three times: the first, after LMA insertion; the second, 10 min after LMA insertion; and the third, before extubation. The means ± SD were statistically analysed

Postoperative complications, such as mucosal hyperaemia, mucosal damage, blood on the surface of the LMA, coughing, bronchospasm and bloating, were similar between the two groups; only one patient in the ProSeal group had a bronchospasm (Table 6).

**Table 6:** Mucosal damage and complications

	Group P (*n* = 60)	Group S (*n* = 60)	*P*
	n (%)	n (%)	
Mucosal Hyperaemia	3 (5.0)	5 (8.3)	0.717
Mucosal Damage	3 (5.0)	5 (8.3)	0.717
Blood on LMA	3 (5.0)	5 (8.3)	0.717
Complications	5 (8.3)	4 (6.7)	1.000
Cough	3 (5.0)	3 (5.0)	
Bronchospasm	1 (1.7)	0 (0.0)	
Bloating	1 (1.7)	1 (1.7)	

*LMA* laryngeal mask airway; *P* < 0.05 is statistically significant

## Discussion

In paediatric anaesthesiology, the use of LMAs is gaining extensive acceptance, due to the greater risk of perioperative respiratory adverse events when tracheal intubation is used.

In the previous literature, many studies have examined different types of LMAs. These studies were conducted on a wide range of children; however, the studies rarely included infants younger than 1 year of age [2–8]. LMAs have been evaluated in infants in only a few studies [11, 13, 14].

In this study, two different LMAs, the ProSeal and the Supreme, were compared in infants younger than 1 year of age. Compared to the Supreme LMA, ProSeal LMA insertion was shorter, the peak and mean pressures were lower, the oropharyngeal leakage pressure was higher and the mean arterial pressure was statistically lower.

Paediatric LMAs are smaller versions of adult LMAs. The ProSeal LMA is a reusable, second-generation device. An adult size of the ProSeal LMA was introduced in 2000, and its paediatric sizes (1.0–2.5) became available in 2004. The Supreme LMA is a single-use, second-generation device. The Supreme LMA was designed as a rigid, curved airway that combines features of the ProSeal and Fastrach™ airways [4, 5, 7]; however, infants have large tongues, floppy epiglottises and more anterior and higher larynxes than those of adults and older children. Such differences might influence the correct placement of the different LMA models.

Kim et al. compared the i-gel® and Classic LMAs in infants and concluded that the i-gel LMA was easier to insert than the Classic LMA in infants, with no differences in insertion times, fibreoptic views through the device, airway leakage pressures or complications between the devices [11].

Sanket et al. compared the i-gel and ProSeal LMAs and reported that both devices were comparable in effectively securing the airway in infants and older children. They emphasised the security of using a size-1 i-gel or ProSeal LMA [6].

In our study, we compared the ProSeal and Supreme LMAs, which both included a gastric access canal to offer protection against unexpected regurgitation and aspiration.

This study included infants scheduled for minor, lower abdominal surgery, such as inguinal hernia or orchiopexi, allowing for easy correction of any respiratory complications.

Pont et al., in a prospective, randomised, observational study, compared the suitability of a size-1 i-gel LMA with a size-1 Classic LMA in paediatric patients undergoing an elective day procedure. The authors concluded that the size-1 i-gel LMA was less prone to displacement during changes in the patient’s position. Because this study was carried out in a small number of patients, these authors believed that further trials were needed to reach definite conclusions about the two devices [14].

In 2015, Jagannathan et al. reviewed the current literature on newer supraglottic airways [4]. One of the studies reviewed was our study, in which we compared the use of the ProSeal and Supreme LMAs in children [5]; however, in that study, the leakage pressures of the LMAs were not calculated or compared.

The leakage pressure is an important parameter of airway safety and is often used to monitor the quality of the airway seal. High pressures usually indicate high safety. Additionally, an LMA’s cuff pressure could greatly influence the leakage pressure. However, an optimal LMA cuff pressure was not determined in this study. Furthermore, in many paediatric anaesthesiology clinics, the LMA cuff pressure is not routinely measured [11, 12]. In an observational study, the use of ultrasound for the correct placement of LMAs in paediatric patients was investigated; this study concluded that ultrasound was unable to detect the suboptimal depth of the LMA but could potentially serve to accurately detect a rotated LMA [3].

In our study, we used small device sizes of 1 and 1.5, inflated the cuff to a 30–40 cm H_2_O pressure and confirmed successful LMA insertion by observing bilateral chest wall movement and a square capnography waveform. We assume that the softer anatomy of infant airways and the rigid, curved design of the Supreme LMA caused more damage to the oral mucosa and a lower leakage pressure than the ProSeal LMA. We believe that the reason why there were no failures in any LMA insertions in our cases was that all cases were conducted in patients of ASA physical status I with normal airways and were performed by the same experienced anaesthesiologist.

Since the introduction of the first supraglottic airways in the literature, many studies have shown that LMAs cause fewer haemodynamic changes during insertion than an intubation tube [15, 16]. The haemodynamic changes caused by LMA insertion were similar to those caused by other standard airway insertions [17]. In our study, the mean HR of the ProSeal group was statistically lower, both before and after extubation, than that of the Supreme group. Many studies have shown that LMAs reduce the required use of specific anaesthetics during surgery, especially neuromuscular blockers [10, 18]. However, many anaesthesiologists prefer to use tracheal intubation in infants due to airway security concerns. Therefore, there is a need to conduct LMA insertion studies in infants, and we designed our study using a relatively large number of infants.

There are some limitations of our study. First, all LMAs were inserted by the same experienced anaesthetist; therefore, it might not be possible to generalise the results to more inexperienced practitioners. Second, our study was conducted on infants of ASA physical status I with normal airways. Finally, we could not confirm the position of the LMAs by fibreoptic laryngoscopy.

## Conclusion

The results of the present study indicate that the ProSeal LMA is superior to the Supreme LMA for use in infants due to its ease of insertion, high oropharyngeal leakage pressure and fewer induced haemodynamic changes.

## Abbreviations

ASA: American Society of Anesthesiologists
BIS: Bispectral index
EtCO_2_: End tidal CO_2_
HR: Heart rate
LMA: Laryngeal mask airway
Min: Minutes
SpO_2_: Oxygen saturation
